# Mapping Soil Properties of Africa at 250 m Resolution: Random Forests Significantly Improve Current Predictions

**DOI:** 10.1371/journal.pone.0125814

**Published:** 2015-06-25

**Authors:** Tomislav Hengl, Gerard B. M. Heuvelink, Bas Kempen, Johan G. B. Leenaars, Markus G. Walsh, Keith D. Shepherd, Andrew Sila, Robert A. MacMillan, Jorge Mendes de Jesus, Lulseged Tamene, Jérôme E. Tondoh

**Affiliations:** 1 ISRIC—World Soil Information, Wageningen, the Netherlands; 2 The Earth Institute, Columbia University, USA / Selian Agricultural Research Inst., Arusha, Tanzania; 3 World Agroforestry Centre, Nairobi, Kenya; 4 LandMapper Environmental Solutions Inc., Edmonton, Canada; 5 International Center for Tropical Agriculture, Lilongwe, Malawi

## Abstract

80% of arable land in Africa has low soil fertility and suffers from physical soil problems. Additionally, significant amounts of nutrients are lost every year due to unsustainable soil management practices. This is partially the result of insufficient use of soil management knowledge. To help bridge the soil information gap in Africa, the Africa Soil Information Service (AfSIS) project was established in 2008. Over the period 2008–2014, the AfSIS project compiled two point data sets: the Africa Soil Profiles (legacy) database and the AfSIS Sentinel Site database. These data sets contain over 28 thousand sampling locations and represent the most comprehensive soil sample data sets of the African continent to date. Utilizing these point data sets in combination with a large number of covariates, we have generated a series of spatial predictions of soil properties relevant to the agricultural management—organic carbon, pH, sand, silt and clay fractions, bulk density, cation-exchange capacity, total nitrogen, exchangeable acidity, Al content and exchangeable bases (Ca, K, Mg, Na). We specifically investigate differences between two predictive approaches: random forests and linear regression. Results of 5-fold cross-validation demonstrate that the random forests algorithm consistently outperforms the linear regression algorithm, with average decreases of 15–75% in Root Mean Squared Error (*RMSE*) across soil properties and depths. Fitting and running random forests models takes an order of magnitude more time and the modelling success is sensitive to artifacts in the input data, but as long as quality-controlled point data are provided, an increase in soil mapping accuracy can be expected. Results also indicate that globally predicted soil classes (USDA Soil Taxonomy, especially Alfisols and Mollisols) help improve continental scale soil property mapping, and are among the most important predictors. This indicates a promising potential for transferring pedological knowledge from data rich countries to countries with limited soil data.

## Introduction

The FAO [1] indicates that in the second quarter of the 21st century 80% of increased crop production in developing countries will have to come from agricultural intensification. Successful intensification requires careful ecological implementation and will need to be underpinned by economically viable investment decisions by the various stakeholders in the value chain (in addition to farmers) [2]. In that context, comprehensive, accurate and up-to-date soil information is an essential input into agricultural and ecological decision making models. Soil information can help predict scenario dependent crop yields as well as water and nutrient dynamics. It can also help identify areas at risk of soil degradation and support choosing appropriate preventive and rehabilitative soil management interventions.

For decades soil information in Africa has been fragmented and limited to specific zones of interest [3]. The Alliance for a Green Revolution in Africa (AGRA) has estimated that up to 80% of arable land in sub-Saharan Africa has low soil fertility and suffers from physical soil problems [4]. At the same time, significant amounts of soil nutrients are lost every year due to inappropriate or unsustainable soil management practices such as intensification (shortening of fallow periods) without adequate nutrient supplements [4]. The Montpellier Panel [5] has estimated that the economic loss in Africa due to poverty, climate change, population pressures and inadequate farming techniques is about $68 billion USD per year. The status of soil conditions contributes importantly to these causes.

Dewitte et al. [6] recently produced a harmonized soil polygon map of Africa at scales varying between 1:1M and 1:5M, known as the ‘*Soil Atlas of Africa*’. The resulting map shows the predicted distribution of World Reference Base soil taxa within soil mapping units. Although soil polygon maps can be useful, the focus of contemporary soil mapping is on improving and extending the technology for collecting soil field observations and on automating the generation of (quantitative) high resolution soil property maps [7, 8]. To help bridge the soil information gap in Africa, an international consortium initially coordinated by the International Center for Tropical Agriculture (CIAT) and later by the Columbia Global Centers (Columbia University), established the African Soil Information Service (AfSIS) project [9]. AfSIS builds on recent advances in digital soil mapping, infrared spectroscopy, remote sensing, (geo)statistics, and integrated soil fertility management in order to improve the way that soils are evaluated, mapped, and monitored, whilst significantly reducing the costs to do so [10, 11]. In that context, a major focus of the AfSIS project is on producing continental and country-wide layers of gridded maps of the spatial distribution of soil properties that may be used to support the selection of appropriate land use options and the optimization of soil management practices.

The spatial resolution of soil mapping is also growing in importance. In order to provide usable soil information for detailed planning, soil maps are required at increasingly finer spatial resolution [12]. Coarse resolution polygon-based soil maps, with an average size of delineations of tens to hundreds of square kilometers, are of limited use for detailed spatial planning at a regional scale and are not capable of supporting activities such as precision agriculture and/or smallholder farmer applications. In comparison to other data types, there are now several global data sets of land cover available at a 30 m spatial resolution [13–15]. Although such detailed resolutions are not yet realistic within the domain of global soil mapping, due to computational and data volume constraints, soil mappers are pushing the boundaries by producing predictions at increasingly fine resolutions for larger and larger areas [16–20].

Here, we present results of recent efforts to improve both the resolution and accuracy of the initial predictions of soil properties created at 1 km spatial resolution [21]. We describe a system for downscaling existing predictions to a 250 m resolution through the use of improved modelling algorithms (random forests). As input data we use the largest current compilation of soil profile and soil point observations for Africa, prepared by the AfSIS project, in conjunction with a large repository of remote sensing based images of explanatory environmental variables with both continental and global extents. We focus on a mapping framework that allows us to produce improved spatial predictions from existing data without additional large investments.

Readers interested in obtaining the input data, output maps and processing scripts in this paper are referred to the AfSIS project website at http://africasoils.net. The output prediction maps of soil properties for Africa at 250 m resolution are referred to as the ‘AfSoilGrids250m’ product and are available for download via http://www.isric.org/data/AfSoilGrids250m.

## Materials and Methods

### Automated soil mapping

The emerging ‘*digital*’ approach to soil mapping is data driven and uses statistical methods and information technology to predict soil properties from soil point observations and correlated, spatially exhaustive environmental variables [8, 22]. This approach benefits from new soil measurement technologies such as soil spectroscopy [23, 24] and newly available global data layers, especially those that are free and publicly distributed such as MODIS products [25], ASTER and Landsat images and SRTM DEM [26].

Soil mapping processes are also increasingly automated, which is mainly due to advances in software for statistical computing and growing processing speed and computing capacity. Fully automated geostatistical mapping, i.e. generation of spatial predictions with little to no human interaction, is today a growing field of geoinformation science [21, 27, 28]. Some key advantages of using automated soil mapping versus more conventional, traditional expert-based soil mapping are [29, 30]:

1. All rules required to produce outputs are formalized. The whole procedure is documented (the statistical model and associated computer script), enabling reproducible research.
2. Predicted surfaces can make use of various information sources and can be optimized relative to all available quantitative point and covariate data.
3. There is more flexibility in terms of the spatial extent, resolution and support of requested maps.
4. Automated mapping is more cost-effective: once the system is operational, maintenance and production of updates are an order of magnitude faster and cheaper. Consequently, prediction maps can be updated and improved at shorter and shorter time intervals.
5. Spatial prediction models typically provide quantitative measures of prediction uncertainty (for each prediction location), which are often not provided in the case of conventional soil mapping.

A disadvantage of automated soil mapping is that many statistical and machine learning techniques are sensitive to errors and inconsistencies in input data. A few typos, misaligned spatial coordinates or misspecified models can create serious artifacts and reduce prediction accuracy, more so than with traditional methods. Also, fitting models using large and complex data sets can be time consuming and selection of the ‘*best*’ model is often problematic. Explicit incorporation of conceptual pedological (expert) knowledge, which can be important for prediction in new situations to address the above issues, can be challenging as well.

### Regression-kriging (RK)

At the heart of any geostatistical mapping algorithm is a geostatistical model from which prediction equations are implemented using a programming language [31]. A common framework for generating spatial predictions with soil data is the regression-kriging (RK) framework [32, 33]. In general terms, RK takes the approach:

$$\begin{array}{c} \text{prediction}\;=\;\begin{array}{c} \text{trend}\;\text{predicted}\\ \text{using}\;\text{regression} \end{array}\;+\;\begin{array}{c} \text{residual}\;\text{predicted}\\ \text{using}\;\text{kriging} \end{array} \end{array}$$ (1)

For example, in the case of linear RK, predictions are generated using [33]:

$$\begin{array}{c} \hat{z}\left(s_{0}\right)=x_{0}^{T}\cdot \hat{\beta }+\lambda^{T}\cdot \left(z-X\cdot \hat{\beta }\right) \end{array}$$ (2)

where **z** is a vector of soil observations at *n* sampling locations, **x**_**0**_ is a vector of *p* predictors (or covariates) at the prediction location **s**_**0**_, $\hat{\beta }$ is the vector of estimated regression coefficients, **X** is a matrix of predictors at the *n* sampling locations, and **λ** is a vector of *n* kriging weights used to interpolate the residuals. The regression-kriging model assumes that the residuals are generated from a normally distributed, second-order stationarity random process—i.e. a random process that has a constant mean and variance, and a spatial correlation that only depends on the separation distance between locations and not on the locations themselves [34].

### Random forests RK model

To allow for more complex soil-environment relationships the linear regression model (Eq 1) can be replaced with ‘*machine learning*’ algorithms. Common machine learning algorithms are: artificial neural networks, support vector machines, classification and regression trees, and random forests [31, 35]. In this paper we specifically evaluate the applicability of the random forests algorithm [36] for soil mapping. This is for two main reasons. First, it has been proven in numerous studies [31, 37, 38] that the random forests algorithm can outperform linear regression. Second, unlike linear regression, random forests has no requirements considering the probability distribution of the target variable and can fit complex non-linear relationships in *p* + 1-dimensional space (where *p* is the number of covariates).

A limitation of using random forests however, is that the model is usually only effective within the range in covariate values exhibited by the training data. Statinikov et al. [39] have shown that random forests may also over-fit data sets that are particularly noisy. Also, model fitting and generation of predictions using random forests is orders of magnitude more time-consuming than linear regression, especially when working with a large number of covariates.

Random forests RK model, including a discussion of comparisons between Mean Error (*ME*) and the Root Mean Squared Error between two spatial prediction algorithms, is explained in greater detail in the Supporting Information (S1 Regression-kriging in R using the Meuse data set).

### Input data: soil profile observations and soil samples

For the purpose of building predictive models and generating soil property maps for Africa at 250 m spatial resolution, we merged two pan-African soil sample data sets (Table 1):

1. Africa Soil Profiles (AfSP) database—a compilation of more than 18,000 legacy soil profiles (ca. 75,000 soil horizons) collected in the past 25+ years by numerous international and national governmental or public organisations and research groups and which, until recently, were typically only available as printed soil survey reports, i.e. only as a paper copy [40],
2. AfSIS Sentinel Site (AfSS) database—containing observations at ca. 9,000 locations (ca. 19,000 soil layers) collected by the AfSIS project during the period 2008–2012 [41].

**Table 1:** List of soil properties of interest. AfSP = Africa Soil Profiles database, AfSS = AfSIS Sentinel Site database. Range was derived as the symmetric 99% quantile range based on observed data. Number of depths column indicates number of output prediction depths e.g.: 6 depths (0–5 cm, 5–15 cm, 15–30 cm, 30–60 cm, 60–100 cm and 100–200 cm) or 2 depths (0–20 cm, 20–50 cm).

			**sample size**			
**GSIF code**	**Variable name**	**Units**	**AfSP**	**AfSS**	**Range**	**Depths**
ORCDRC	Soil organic carbon concentration (fine earth)	g kg^−1^	45956	18054	0.9–42 ‰	6
PHIHOX	Soil pH in H2O	–	50403	18055	4.4–8.7	6
SNDPPT	Soil texture fraction sand	kg kg^−1^	54170	1408	7–94%	6
SLTPPT	Soil texture fraction silt	kg kg^−1^	54164	0	1–47%	6
CLYPPT	Soil texture fraction clay	kg kg^−1^	54167	0	3–73%	6
BLD	Bulk density (fine earth)	t m^−3^	8732	0	0.9–1.9	6
CEC	Cation Exchange Capacity (fine earth)	cmol+/kg	47875	0	1.2–57	6
NTO	Total nitrogen	g kg^−1^	50997	18054	0.1–3.1 ‰	2
ALUM3S	Exchangeable Aluminium	mg kg^−1^ (ppm)	4305	18055	150–1800	2
EACKCL	Exchangeable acidity	cmol+/kg	24242	0	0–6.4	6
ECAX	Exchangeable Calcium	cmol+/kg	47103	18055	0.1–46	2
EXKX	Exchangeable Potassium	cmol+/kg	46463	18055	0.01–2.4	2
EMGX	Exchangeable Magnesium	cmol+/kg	45206	18055	0.04–15	2
ENAX	Exchangeable Sodium	cmol+/kg	41572	1414	0–8.3	2
EXBX	Sum of exchangeable bases	cmol+/kg	46215	18055	0.31–66	6

The AfSP database contains a diverse collection of field and laboratory observations, usually collected per pedogenetic horizon up to 2 m depth. The data from the original soil survey reports (1950–2014) were collated, standardized and where possible, harmonized, quality controlled, and integrated into a consistent database [40].

The AfSIS Sentinel Site database, one of the major deliverables of the first phase of the AfSIS project (2008–2013), was generated using a consistent soil sampling design. The soil samples from the sentinel site network were collected using a multilevel sampling scheme: a number of ‘*Sentinel Sites*’ (10 × 10 kilometers in size) were first selected across the whole continent (60 sites in 2012) using stratified random sampling. Stratification was done based on the major Koeppen-Geiger climate zones of Africa, excluding true deserts and, for security reasons, specific African countries. Within each of the 60 Sentinel Sites, AfSIS field teams sampled 16 ‘*Sampling Clusters*’ consisting of 10 randomly located circular ‘*Sampling Plots*’ (covering 1000 m). At each sampling plot, composite soil samples (a centroid composed of four points) were taken at two depths (0–20 and 20–50 cm), so that, in summary, the AfSIS Sentinel Site database consists of:

- 60 Sentinel Sites,
- 16 Sampling Clusters per Sentinel Site,
- 10 Sampling Plots per Sampling Cluster,
- 2 composite soil samples (0–20 and 20–50 cm) per Sampling Plot.

**Fig 1:**
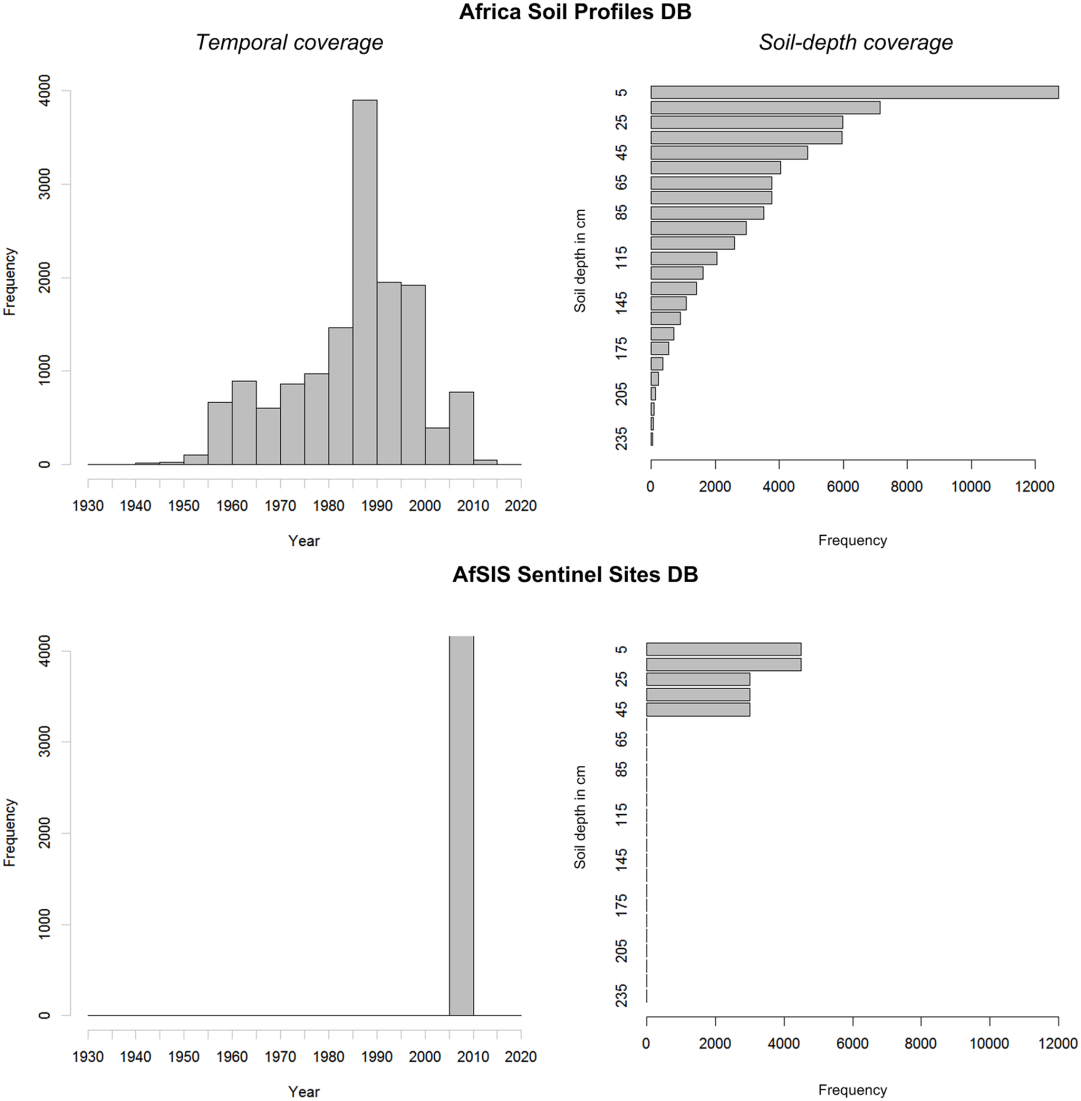
Temporal and soil-depth coverage of the Africa Soil Profiles and AfSIS Sentinel Site databases. See also Table 1.

This sums to about 19,200 samples / layers collected at about 9,600 unique locations (Fig 1). The sampling and analysis protocols used to produce the AfSIS Sentinel Site database are described in detail in Vågen et al. [41, 42].

All soil samples of the AfSS database were analysed using soil diffuse reflectance spectroscopy, while only 10% were additionally analysed using laboratory methods (cf. ‘*wet-chemistry*’). Next all soil spectral signatures were translated to soil properties by fitting soil spectroscopy calibration models to the set of reference soil samples for which both spectral signatures and wet chemistry observations were available. For more details about the calibration models and their accuracy, refer to the soil.spec package for R [43].

**Fig 2:**
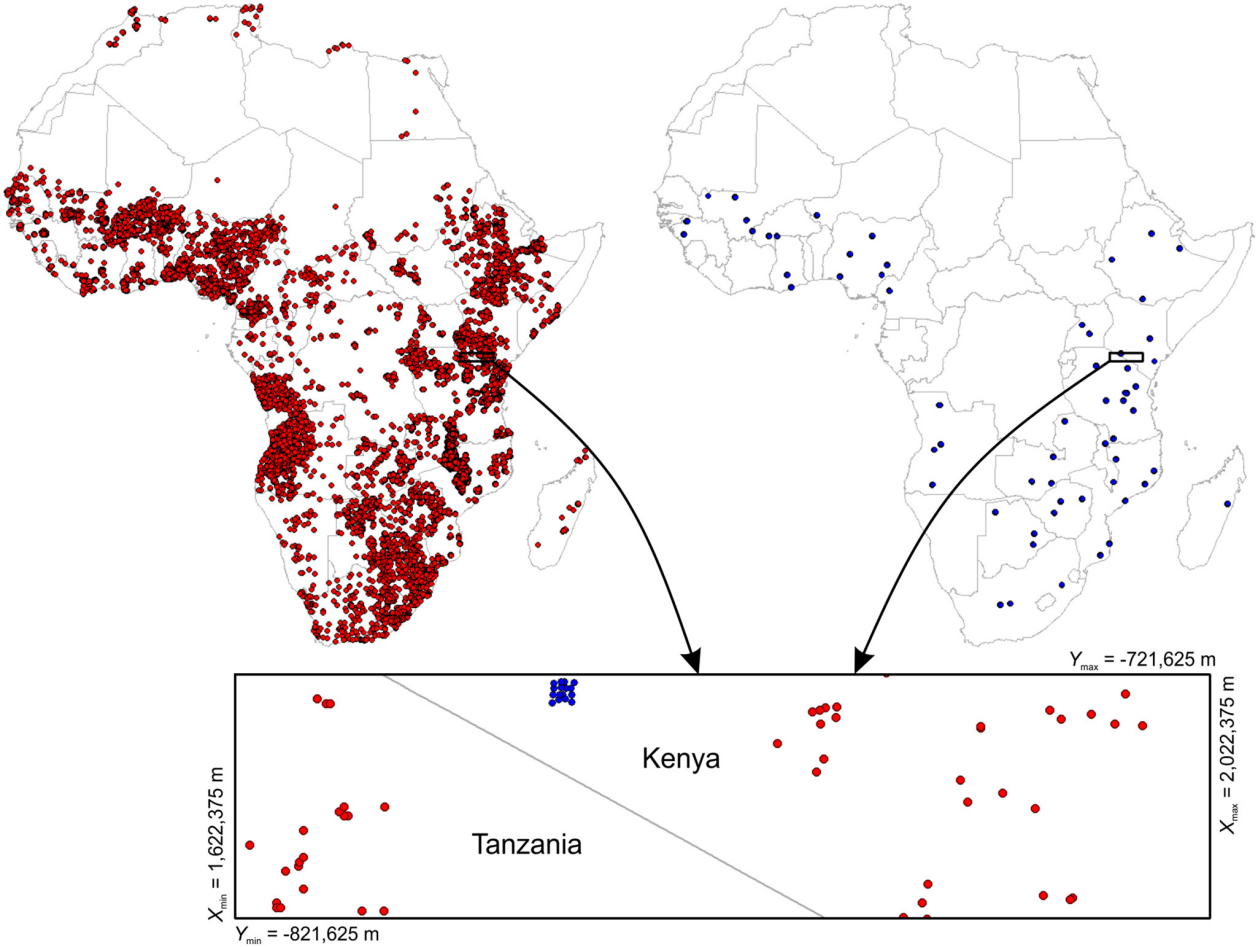
Distribution of soil samples in Africa used to build spatial predictive models. (left) legacy soil profile observations (Africa Soil Profiles database) showing ca. 18.5 thousand locations [40], and (right) AfSIS Sentinel Sites showing ca. 9.5 thousand locations, but which are clustered at 60 sentinel sites [41]. Zoom-in on the example area (100 by 400 km) shown further in Figs 6 and 9. Coordinates in the Lambert Azimuthal Equal Area projection (WGS84 ellipsoid) with latitude at projection center = 5°, longitude at projection center = 20°.

The distribution of the ca. 28,000 sampling locations of the two data sets is shown in Fig 2. Note that neither of the data sets have ideal properties for geostatistical analyses. For example, the Africa Soil Profiles Database is a compilation of soil samples from a multitude of different soil survey projects and analysed in various laboratories following a diversity of soil survey methods. Only limited harmonization has been applied so far and measurement and location error is in many cases unknown, but could be substantial (the estimated average location error radius is between 100–1800 m). The African Sentinel Sites data on the other hand, were analysed using consistent, standardized laboratory methods, hence they are nominally more suitable for soil predictive modelling because no harmonization is required. However, the sampling points in AfSS database are clustered around 60 (10 × 10 kilometers) sentinel sites and characterize only 0–50 cm soil depths (as compared to the legacy soil profiles that cover also depths > 50 cm).

### Environmental covariates

In addition to the point data we also used a large collection of raster images as covariate layers to fit predictive models. These come from four main sources:

1. MODIS products at 250 m resolution—We used the Mid-infrared (MIR) Reflectance (Band 7) Long-Term and Monthly Averages and Enhanced Vegetation Index (EVI) Long-Term and Monthly Averages (MOD13Q1 product) [25]. These layers were prepared for the purpose of the AfSIS project by the Earth Institute at Columbia University and are available for download at http://africasoils.net/data/datasets.
2. SRTM DEM v4.1 based covariates—We used elevation, slope and SAGA GIS Topographic Wetness Index (TWI), all derived at 250 m resolution [26].
3. GlobeLand30—We used the fraction of coverage for ten land use classes from the global land cover map for 2010 [15], which were resampled from 30 m to 250 m resolution in SAGA GIS. These layers were also used to determine the soil mask i.e. areas of interest for soil mapping.
4. SoilGrids1km—We used 1 km–resolution predictions of soil properties and classes produced previously using global models [21]. These were first downscaled to 250 m resolution by bicubic resampling, as implemented in the SAGA GIS software [44].

Covariates were selected to represent the major soil forming processes and surface characteristics. The MODIS MIR and EVI products represent spectral signatures of different surface materials and vegetation types. Elevation, slope and Topographic Wetness Index are common soil covariates representing landscape morphology and erosion / deposition processes. SoilGrids1km predictions were based on analysis of a large number of environmental covariates, such as climatic images, lithology and land cover maps, Harmonized World Soil Database mapping units and similar [21].

### Soil mask layer

We make predictions only for areas with vegetation cover (about 21 million square-kilometers), i.e. all deserts and shifting sand areas were excluded. We derived the soil mask map for Africa by using the GlobeLand30 data set [15] whereby pixels that had > 10% of bare land (class 90 in GlobeLand30) and/or > 30% of water cover (class 60 in GlobeLand30) were removed from the mask. For permanent deserts we do not provide any predictions but advise users to fill in those areas with expert-based estimates of soil properties, e.g. sand content > 95% and organic carbon content < 1 permilles.

### Prediction models

We implemented two soil mapping frameworks for producing spatial predictions of soil properties. In the first framework, we predicted soil properties at 250 m resolution, that had previously been mapped at 1 km using global soil prediction models [21], e.g. soil organic carbon, pH, sand, silt, clay, bulk density, Cation Exchange Capacity and depth to bedrock. This is basically a downscaling framework in which the global predictions (1 km) are ‘*refined*’ or downscaled using finer resolution covariates (250 m), e.g.:

$$\begin{array}{c} z^{250m}=f\left(z^{1\text{km}},X^{250m},d\right)+\varepsilon \end{array}$$ (3)

where *z* ^250m^ is the target variable, *z* ^1km^ is the globally predicted (SoilGrids1km system) same variable (i.e. same soil property and same depth) at coarser resolution, **X** ^250m^ are the values of local covariates at the finer resolution, *f* is the regression function, *ε* is a spatially auto-correlated residual, which we interpolate using kriging, and *d* is depth. We define for example the model for organic carbon as (in R syntax):

*R> ORCDRC ^~^ m_ORCDRC + af_DEMSRE5a.tif + af_SLPSRE5a + af_TWISRE5a + …*

*+ af_GLC_10_250m.tif + … + af_GLC_100_250m.tif + …*

*+ af_M13EVIA01 + af_M13EVIA02 + … + af_M13RB7A01 + af_M13RB7A02 + …*

*+ af_PC1EVI5a + … + af_PC4EVI5a + altitude*

where m_ORCDRC is the value of organic carbon predicted at 1 km resolution (SoilGrids1km layer) at the depth of sampling ORCDRC; af_DEMSRE5a, af_SLPSRE5a, af_TWISRE5a are elevation, slope and SAGA GIS Topographic Wetness Index derived at 250 m; af_GLC_x0_250m.tif is the fraction of the land cover class from the GlobeLand30 product [15]; af_M13EVIAxx and af_M13RB7Axx are the long-term (2001–2013) standardized values of MODIS EVI and mid-infra red (band 7) products for months January to December; af_PC1-4EVI5a are the 1–4 principal components derived using annual MODIS EVI images (2001–2013), and altitude is soil depth, i.e. a depth at which the target variable has been measured.

The trend model for organic carbon can be fitted using random forests or linear regression, while the residuals can be interpolated using ordinary kriging. In the case of linear RK, predictions using the model Eq (3) are generated by:

$$\begin{array}{c} {\hat{z}}^{250m}\left(s_{0}\right)={\hat{\beta }}_{0}+{\hat{\beta }}_{1}\cdot {\hat{z}}^{1\text{km}}\left(s_{0}\right)+\sum_{j=2}^{p}{\hat{\beta }}_{j}\cdot X_{j}^{250m}\left(s_{0}\right)+\lambda^{T}\cdot \varepsilon \end{array}$$ (4)

where ***λ*** is the vector of kriging weights and ***ε*** is a vector of regression residuals at observation locations.

In the second framework, we consider soil properties that have not yet been mapped previously using global models, such as Al concentration, exchangeable bases Ca, K and Mg. Here, we build models using global soil class maps (i.e. predicted probabilities for each class) from the SoilGrids1km system as global covariate layers:

$$\begin{array}{c} z^{250m}=f\left(\mu^{1\text{km}},X^{250m},d\right)+\varepsilon \end{array}$$ (5)

where ***μ*** ^1km^ is the stack of soil class probabilities predicted globally (SoilGrids1km system). As an example, the model used to predict exchangeable Mg is given in R syntax by:

*R> EMGM3S ^~^ m_TAXOUSDA_Albolls + m_TAXOUSDA_Aqualfs …*

*+ af_DEMSRE5a.tif + af_SLPSRE5a + af_TWISRE5a + …*

*+ af_GLC_10_250m.tif + … + af_GLC_100_250m.tif + …*

*+ af_M13EVIA01 + af_M13EVIA02 + … + af_M13RB7A01 + af_M13RB7A02 + …*

*+ af_PC1EVI5a + … + af_PC4EVI5a + altitude*

where m_TAXOUSDA_x are predicted probabilities of the United States Department of Agriculture (USDA) Soil Taxonomy suborders (67 suborders in total) at 1 km resolution (SoilGrids1km data set). We used the USDA soil classes instead of World Reference Base classes because these were mapped with somewhat greater accuracy in the SoilGrids1km product and because this classification system contains about twice as many classes [21].

Because the main focus of this paper is the comparison between linear regression and random forests, for each property in Table 1 we fit a non-linear regression model using the random forests algorithm as implemented in the randomForest package [45], and a linear regression model using the same formula. For both models we then derived the residuals and fitted residual variograms in 3D using the gstat package.

### Variogram estimation

After the regression fitting, we modelled all variograms using an exponential model with three standard parameters (nugget *c*_0_, partial sill *c*_1_, range parameter *r*):

$$\begin{array}{c} \gamma (h)=\{\begin{array}{ccc} 0 & \text{if} & h=0\\ c_{0}+c_{1}\cdot [1-e^{-(\frac{h}{r})}] & \text{if} & h>0 \end{array}\;h=[h_{x},h_{y},h_{d}] \end{array}$$ (6)

where the scalar ‘*distance*’ *h* is calculated by scaling horizontal and vertical separation distances using three anisotropy parameters:

$$\begin{array}{c} h=\sqrt{{\left(\frac{h_{x}}{a_{x}}\right)}^{2}+{\left(\frac{h_{y}}{a_{y}}\right)}^{2}+{\left(\frac{h_{d}}{a_{d}}\right)}^{2}} \end{array}$$ (7)

**Fig 3:**
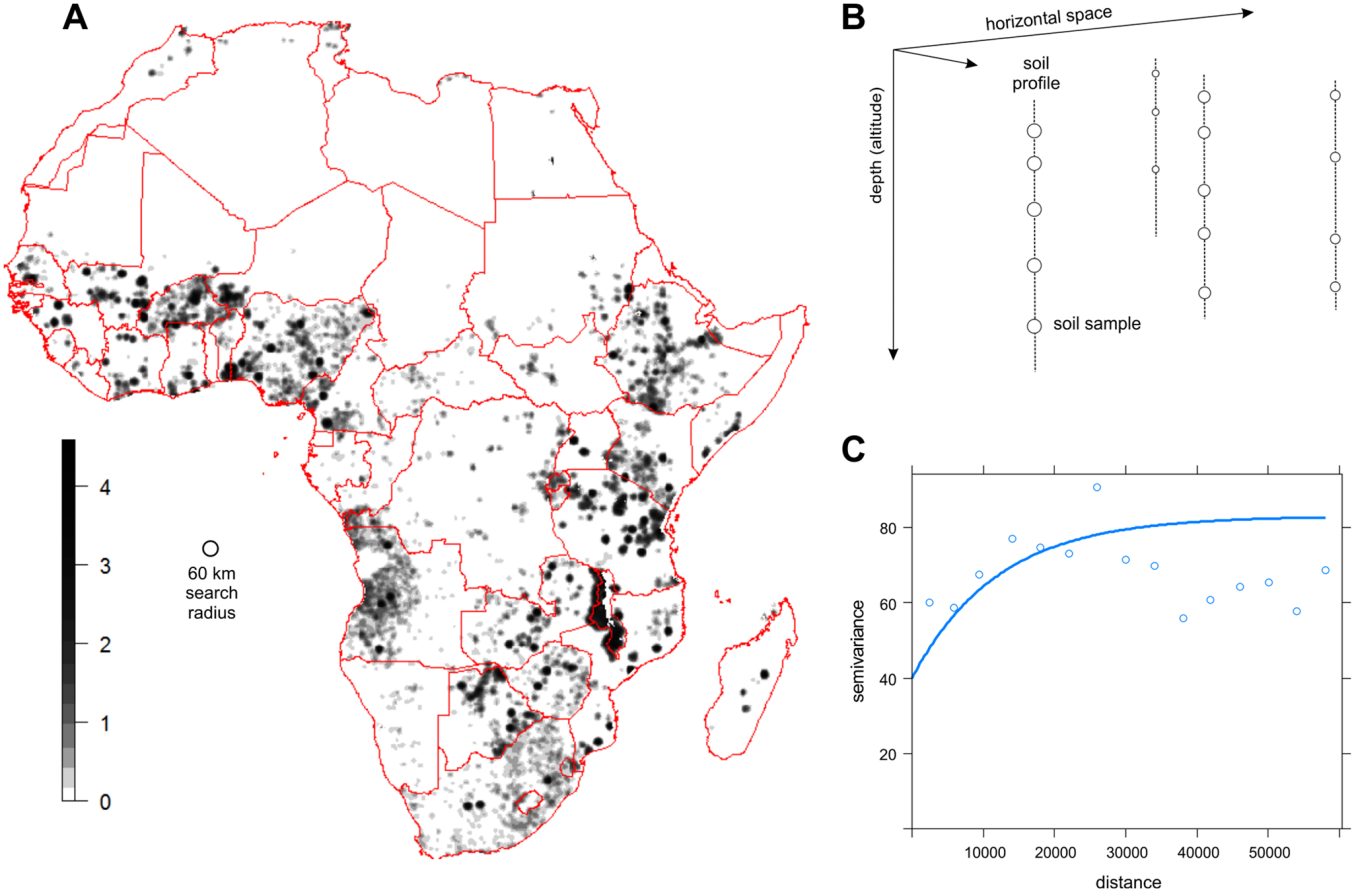
Density of soil observations in Africa showing distinct spatial clustering and an example of residual variography. (A) relative density of soil observations in Africa determined using a kernel smoother displayed in log-scale (input locations shown in Fig 2), (B) 3D sampling locations scheme, (C) example of an exponential variogram fitted for soil organic carbon residuals. In this case the maximum distance of interest for kriging has been set at 60 km.

Typically, in the case of soil data, the anisotropy ratio between horizontal and vertical distances is high—spatial variation observed in a few cm depth change may correspond with several km or more in horizontal space, so that the initial settings of the anisotropy ratio (i.e. the ratio of the horizontal and vertical variogram ranges) are between 3000–8000, for example. Variogram fitting criteria can then be used to optimize the anisotropy parameters. In our case we assumed no horizontal anisotropy and hence assumed *a*_*x*_ = *a*_*y*_ = 1, leaving only *a*_*d*_ to be estimated. Once the anisotropy ratio is obtained, 3D variogram modelling does not meaningfully differ from 2D variogram modelling (e.g. see Fig 3C).

The 3D RK framework explained above can be compared to the approach of Malone et al. [46], who first fit equal-area spline function to estimate the soil properties at a standard depth, and next fit regression and variogram models at each depth. A drawback of the approach by Malone et al. [46], however, is that the separate models for each depth ignore all vertical correlations. In addition, the equal-area spline is not used to model soil-depth relationships but only to estimate the values at standard depths for sampling locations i.e. it is implemented for each soil profile (site) separately. In the 3D RK framework explained above, a single model is used to generate predictions at any location and for any depth, and which takes into account both horizontal and vertical relationships simultaneously. This approach is both easier to implement, and allows for incorporating all (vertical) soil-depth relationships including the spatial correlations.

### Evaluation criteria

We evaluated the performance of each predictive model by calculating the Mean Error (*ME*) and Root Mean Squared Error (*RMSE*) using 5–fold cross-validation. We also calculated the *t*- and *F*-test statistics, as explained in the supporting information, to test if the random forests model outperforms the linear regression model with statistical significance. For each predictive model, we also report the amount of variation at validation points explained by the model, derived as:

$$\begin{array}{c} \Sigma_{\%}=[1-\frac{SSE}{SST}]\;\left[0-100\%\right] \end{array}$$ (8)

where *SSE* is the sum of squared errors at the cross-validation points (i.e. *RMSE* ^2^ ⋅ *n*), and *SST* is the total sum of squares of the original observations. The amount of variation explained by the model is dimensionless and allows for comparing mapping accuracy between different variables (see supporting information). The *t*-test and *F*-test assume that the target variable has a close-to-normal distribution, which means that we run all testing and visualization in the transformed spaces for all soil properties except pH, sand, silt and clay content and bulk density.

In addition to 5–fold cross-validation, we also test predictability of soil properties using legacy soil profile data only (AfSP) vs Sentinel Site (AfSS) data and vice versa—predictability of models fitted using Sentinel Site data vs legacy soil profile data. This comparison is done primarily to identify those soil properties for which the predictability is critically weak when part of the combined data set is completely removed.

### Final predictions

The final RK predictions were produced using a sum of the regression and kriging parts, as indicated in Eq (4). Again, since we work at 250 m resolution, the whole process from data overlay to model fitting and prediction can take several hours; spatial predictions can take weeks of computing. To speed up the processing we used a combination of tiling and parallel processing, as implemented via the snowfall package for R [47]. The approximate processing time per soil property, using the computer specifications mentioned in the discussion section, was:

- spatial overlay and generation of regression matrix: 20–30 min,
- fitting of random forests model and residual variogram: 30–60 min,
- spatial prediction using random forests (using tiling): 100–150 min,
- spatial interpolation of residuals using 3D ordinary kriging: 10–20 min,
- mosaicking and data export to GDAL supported format: 10–15 min.

To speed up 3D kriging of residuals for highly clustered point data, we used an approximation of ordinary kriging implemented via the spline.krige function in the GSIF package [48]. The spline.krige function first adjusts the density of prediction locations by calculating the kernel density of the observation locations, such that areas that are only sparsely sampled (Fig 3A) have a proportionally coarser prediction grid. This yields a ‘*variable*’ grid with fine resolution only there where there are observations nearby. Kriged residuals at the 250 m grid are then derived from those at the variable grid using spline interpolation, as implemented in the SAGA GIS software.

The spline.krige function speeds up the kriging calculations by an order of magnitude, with minimal loss in precision, whilst reducing generation of kriging artifacts. Achieving the right balance between processing time and accuracy gain is especially important considering that we are dealing with large data sets and that most of the detail in spatial patterns comes from the random forest models and not from residual kriging. Our initial testing indicated that, if the parameters of the spline.krige function are set correctly, the loss in precision rarely exceeds 1%.

## Results

### Mapping accuracy

Table 2 shows cross-validation summary statistics comparing linear regression with random forests regression (as implemented in the randomForest package [45]). For all properties, the random forests model yields more accurate predictions than the linear regression model. The *F*-test results in Table 2 show that the variance reduction compared to the linear model is statistically significant in all cases. The relative improvement in mapping accuracy is between 15–75%. Bulk density, aluminum concentration and exchangeable acidity benefit most from replacing linear regression with random forests, while the accuracy gain is smallest for organic carbon and total nitrogen. The percentage of explained variation varies between 40–85% for random forests, and between 10–45% for linear regression.

**Table 2:** Summary statistics for mapping accuracy assessed using 5–fold cross-validation. *ME* is the mean error, *RMSE* the root mean squared error, sg1km are the SoilGrids1km map, rf represents random forest model predictions and lm the linear model predictions (trend model predictions only). The *t*-test evaluates the difference between the mean errors of the rf and lm models with alternative hypothesis that the difference is greater than 0. The *F*-test evaluates the ratio between the residual variances of the rf and lm models with alternative hypothesis that the difference is greater than 1. Σ_%_ indicates amount of variation explained by the prediction models and Δ*RMSE*_%_ indicates improvement in *RMSE* in percentages compared to the lm model. The ‘⋆⋆⋆’ indicates significance at the 99% probability level. For all soil properties except PHIHOX, SNDPPT, SLTPPT, CLYPPT and BLD, the Σ_%_, the *t*-test, and the *F*-test have been calculated in log-transformed space. SP-SS are the predictions at Sentinel Sites produced using models fitted from AfSP data, SS-SP are the predictions at legacy soil profiles produced using AfSS data. See Table 1 for more details.

	sg1km	lm	rf		sg1km	lm	rf		rf		SP-SS	SS-SP
**GSIF code**	***ME***	***ME***	***ME***	***t*-test**	***RMSE***	***RMSE***	***RMSE***	***F*-test**	**Σ_%_**	**Δ*RMSE*_%_**	***RMSE***	***RMSE***
ORCDRC	1.429	0.113	0.308	1.000	13.0	12.2	10.6	0.000^⋆⋆⋆^	61.3	+15.1	11.4	14.4
PHIHOX	-0.063	0.002	0.006	0.985	0.933	0.886	0.673	0.000^⋆⋆⋆^	66.9	+31.6	0.69	1.01
SNDPPT	-1.117	-0.035	-0.066	0.209	23.0	21.2	15.9	0.000^⋆⋆⋆^	61.1	+33.3	27.2	29.7
SLTPPT	-2.396	0.040	0.190	1.000	11.9	10.9	8.31	0.000^⋆⋆⋆^	56.1	+31.2	NA	NA
CLYPPT	-3.087	0.005	0.183	1.000	18.1	17.1	13.7	0.000^⋆⋆⋆^	52.4	+24.8	NA	NA
BLD	0.007	0.000	0.002	0.749	0.227	0.213	0.141	0.000^⋆⋆⋆^	70.4	+51.1	NA	NA
CEC	1.114	-0.050	0.152	0.000^⋆⋆⋆^	11.4	11.0	7.92	0.000^⋆⋆⋆^	66.3	+38.9	NA	NA
NTO	NA	0.001	0.015	0.000^⋆⋆⋆^	NA	0.818	0.691	0.000^⋆⋆⋆^	61.0	+18.4	0.688	0.977
ALUM3S	NA	0.016	0.535	0.000^⋆⋆⋆^	NA	279	160	0.000^⋆⋆⋆^	86.3	+74.4	912	NA
EACKCL	NA	-0.004	0.033	0.000^⋆⋆⋆^	NA	2.14	1.30	0.000^⋆⋆⋆^	77.3	+64.6	NA	NA
ECAX	NA	-0.027	0.295	1.000	NA	16.2	12.7	0.000^⋆⋆⋆^	67.2	+27.6	27.9	18.4
EXKX	NA	-0.002	0.017	0.817	NA	0.740	0.599	0.000^⋆⋆⋆^	58.6	+23.5	0.435	0.925
EMGX	NA	0.016	0.083	0.000^⋆⋆⋆^	NA	3.58	2.52	0.000^⋆⋆⋆^	66.0	+42.1	2.40	4.41
ENAX	NA	0.001	0.085	0.000^⋆⋆⋆^	NA	4.25	3.61	0.000^⋆⋆⋆^	46.7	+17.7	5.63	5.82
EXBX	NA	0.022	0.314	1.000	NA	15.5	11.0	0.000^⋆⋆⋆^	68.8	+40.9	20.9	17.8

Note the negative *R* ^2^ value of the linear regression model for exchangeable sodium (ENAX), which means that the model performs worse than using the mean of all observations as predictor; for the random forests model, exchangeable sodium is the soil property that is the least well predicted. Also note that the SoilGrids1km maps are not available for the soil nutrients, hence missing rows in Table 2.

For six properties the random forests model has a significantly smaller *ME* than the linear model. For the other properties, there is either no significant difference in mean error or the linear regression model has a smaller *ME* than the random forests model. The improvement in mapping accuracy when comparing random forests with linear regression is especially evident for pH, bulk density, total Al, and most of the exchangeable bases (especially Mg and Ca). For all these variables both *F*- and *t*-tests show statistically significant improvements, which means that both the residual error and the mean error are significantly reduced.

The column Δ*RMSE*_%_ in Table 2 shows the relative improvement in accuracy of the linear regression model compared to the SoilGrids1km soil property maps. These statistics show that refining the SoilGrids1km maps with finer-resolution (250 m) DEM and satellite imagery improves mapping accuracy. In addition, the mean errors of the linear model predictions are smaller than those of the SoilGrids1km maps, which show considerable bias for most properties. The validation results also show that the gain in prediction accuracy resulting from using a non-linear model instead of linear model (which is roughly the difference between the Δ*RMSE*_%_ statistics) is larger than the gain resulting from refining relatively coarse resolution predictions (1 km) with fine-resolution (250 m) covariate information.

Columns SP-SS and SS-SP in Table 2 show that, in principle, legacy soil profile data have shown to be more useful than the sentinel site data for mapping soil organic carbon, pH, total nitrogen, exchangeable K and Mg—the results of cross-validation using only Sentinel Site data for these variables are consistent with the 5-fold cross-validation. Spatial predictions using Sentinel site data as the only training data seem to result in better predictions only for exchangeable Ca and total exchangeable bases. These results make sense as the number of legacy soil profile observations is about 5–6 times greater than for the Sentinel Sites. In addition, the legacy observations are also more broadly distributed across Africa.

**Fig 4:**
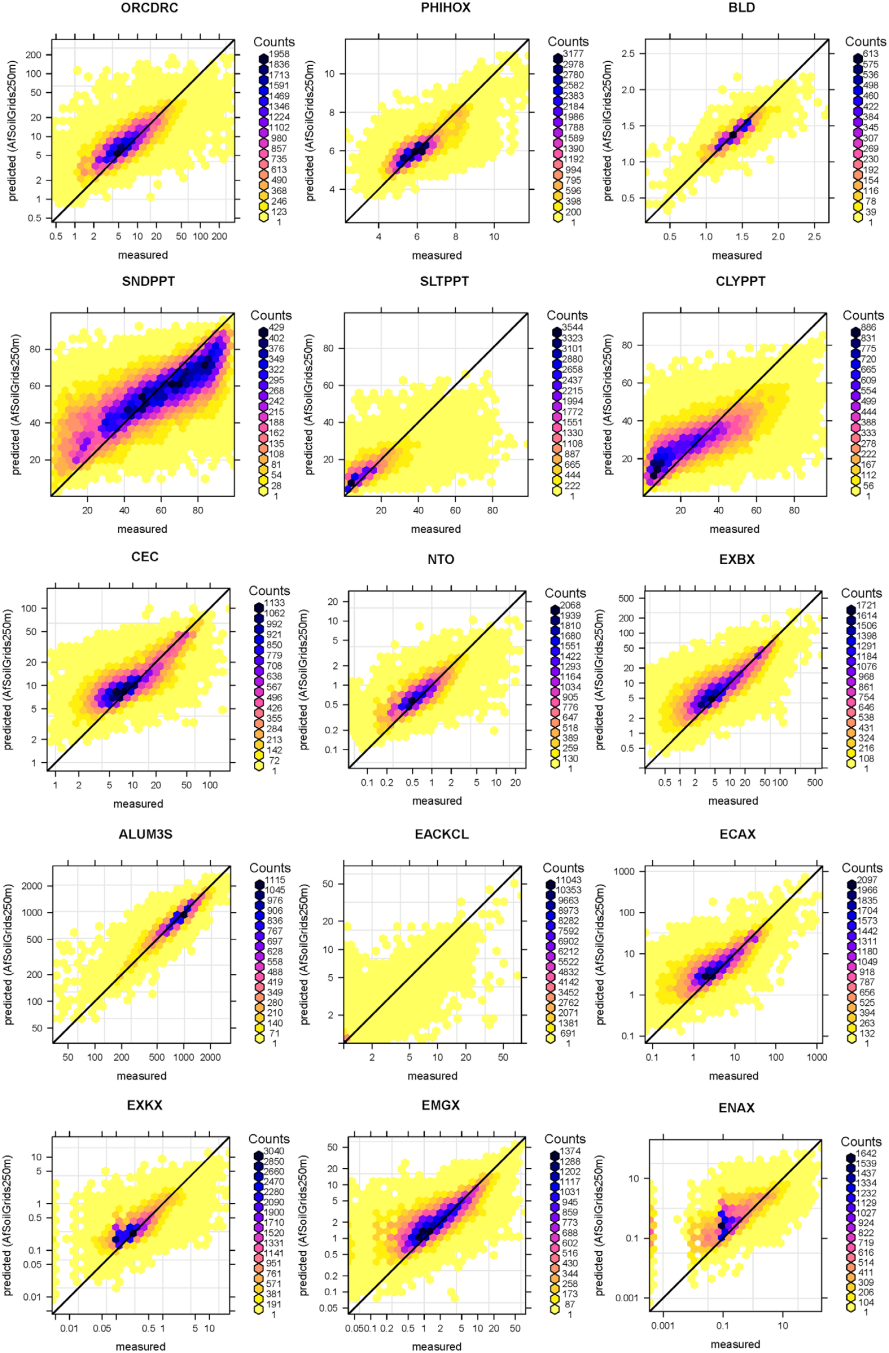
Scatter plots of 5-fold cross-validation errors for soil organic carbon (ORCDRC), pH in water (PHIHOX), bulk density (BLD), soil texture fractions (SNDPPT, SLTPPT and CLYPPT), Cation Exchange Capacity (CEC), total nitrogen (NTO), exchangeable bases (EXBX), total Aluminium (ALUM3S), exchangeable acidity (EACKCL), exchangeable Potassium (EXKX), exchangeable Calcium (ECAX) and exchangeable Magnesium (EMGX). See also Table 2.

Fig 4 shows cross-validation plots for a selection of soil properties obtained with the random forest RK model using local and global covariates. In all cases there seems to be a reasonable match between observed and predicted, although in some cases there is still quite some scatter around the 1:1 line.

### Best predictors

**Fig 5:**
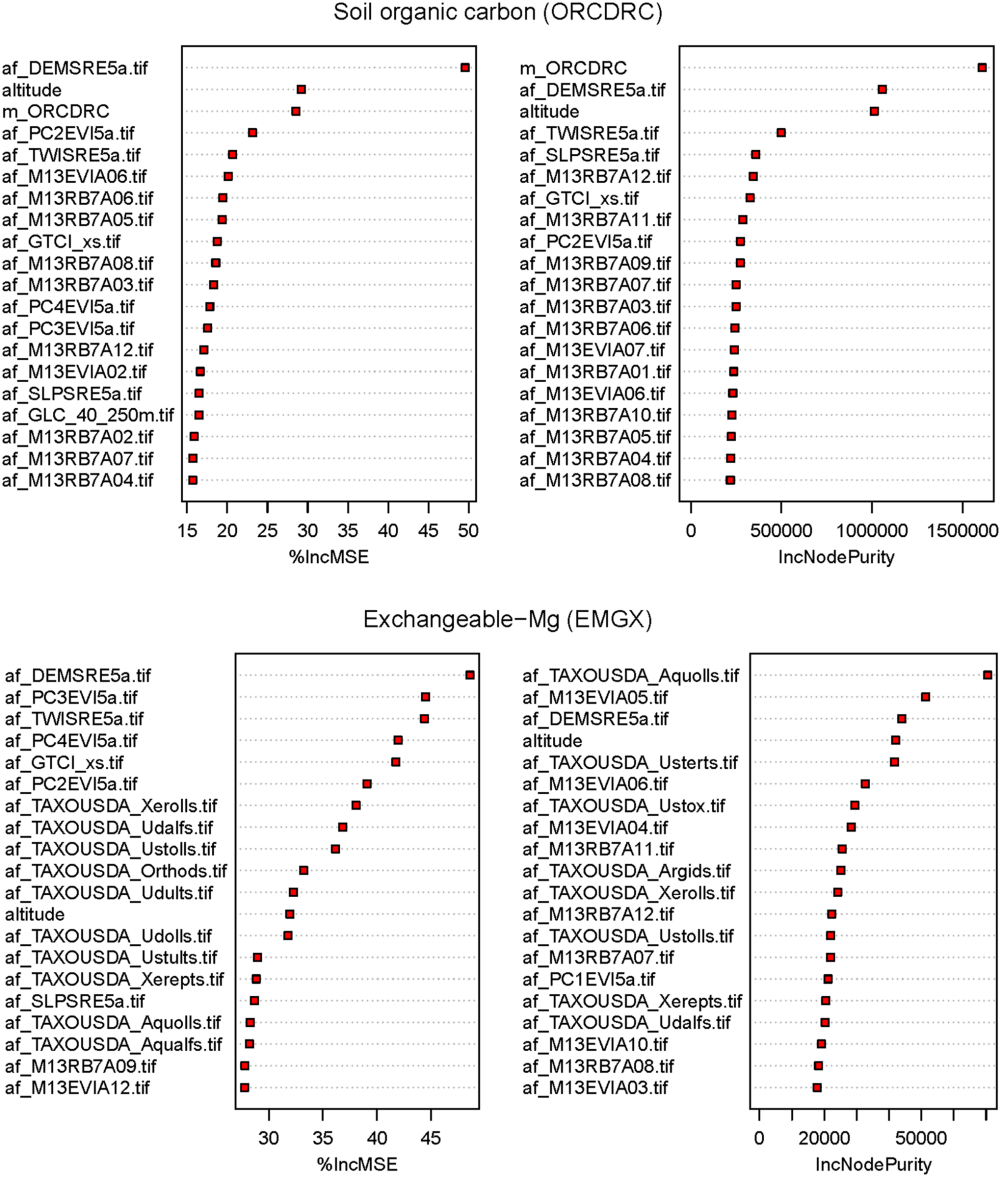
Importance plots for soil organic carbon and exchangeable Magnesium. derived using the varImpPlot function available in the randomForest package [45]. m_TAXOUSDA_x are the predicted SoilGrids1km Soil Taxonomy suborders class probabilities, af_DEMSRE5a, af_SLPSRE5a, af_TWISRE5a are the elevation, slope and SAGA GIS Topographic Wetness Index derived at 250 m resolution, af_M13EVIAxx and af_M13RB7Axx are the long-term (2001–2013) standardized values of MODIS EVI and mid-infra red (band 7) products for months January to December, and af_PC1–4EVI5a are the first four principal components derived from annual MODIS EVI images (2001–2013).

Fig 5 shows ‘*importance plots*’ produced by the randomForest package, which portray the importance of different predictor variables. Not surprisingly, the most important covariates for mapping soil properties that have already been mapped at 1 km globally are the SoilGrids1km layers. This confirms that the method derived from Eq (3) is a useful way to downscale soil property predictions from 1 km (global predictions) to 250 m resolution (African continent). While much of the variation in target soil properties is explained by the global maps of these same soil properties, at 250 m resolution, local covariates still help refine spatial detail and improve prediction accuracy. This appears mainly to be attributable to more detailed information about landform and vegetation cover contained in the local covariates.

For soil properties that were not previously mapped at 1 km resolution, randomForest usually identifies (through the variable importance measure) occurrence probabilities of soil types (especially Alfisols and Mollisols) as the best or second-best predictors of exchangeable bases (see for example Fig 5, right). This fits well with pedological knowledge: Alfisols have relatively high (at least 35%) base saturation, meaning calcium, magnesium, potassium and/or sodium are relatively abundant on the exchange complex. Note that the predicted Aqoulls (Fig 5) come maybe as less probable class in Africa (probabilities < 15%), but it appears that Aquolls-ness of the environmental conditions helps predict some macro-nutrients quite distinctly.

### Final predictions

**Fig 6:**
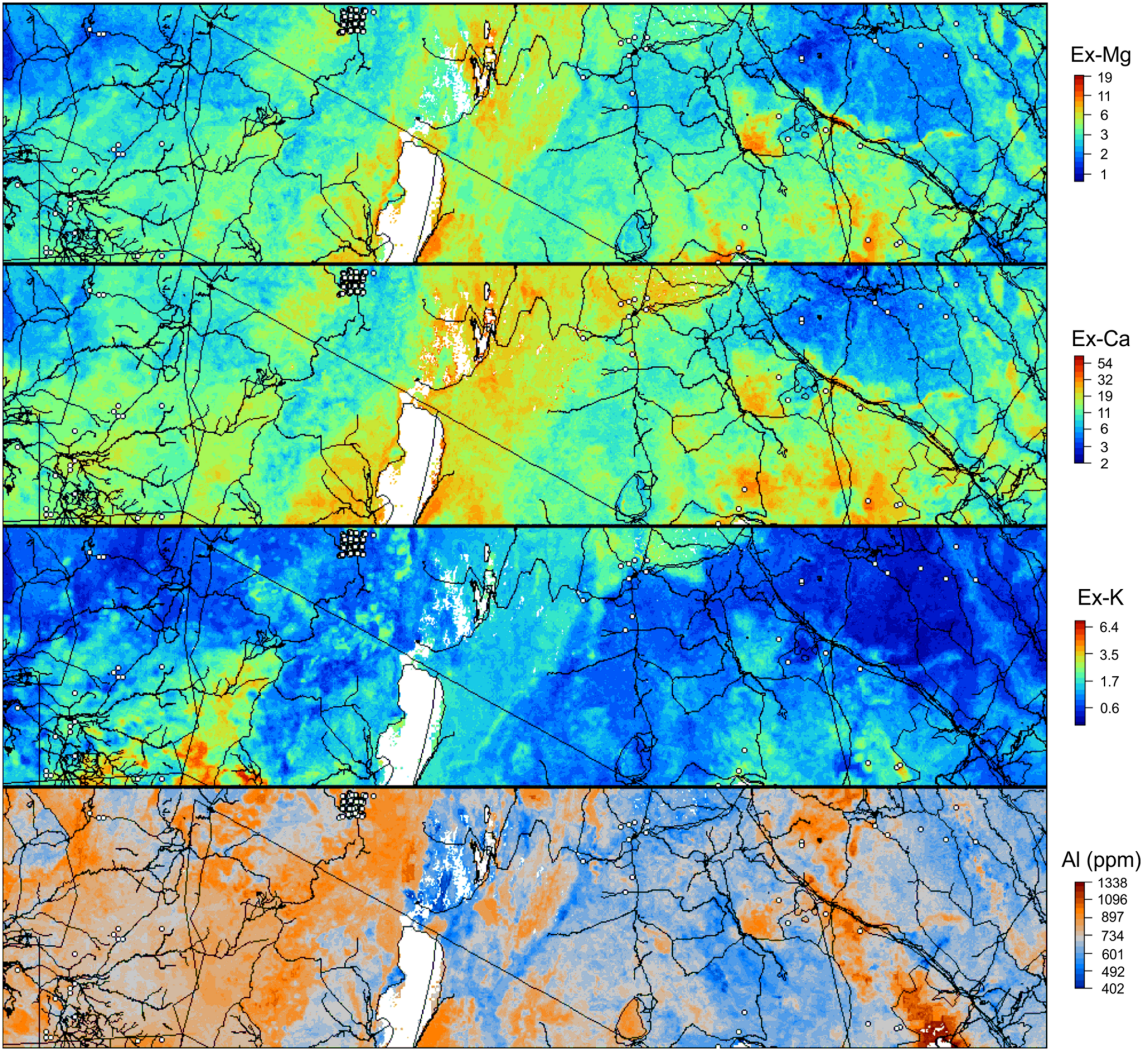
Predicted exchangeable Mg, Ca and K (in cmol+/kg) and Al concentration (ppm) using random forests RK model: zoom-in on the example area from Fig 2. Vector lines data source: OpenStreetMap.

**Fig 7:**
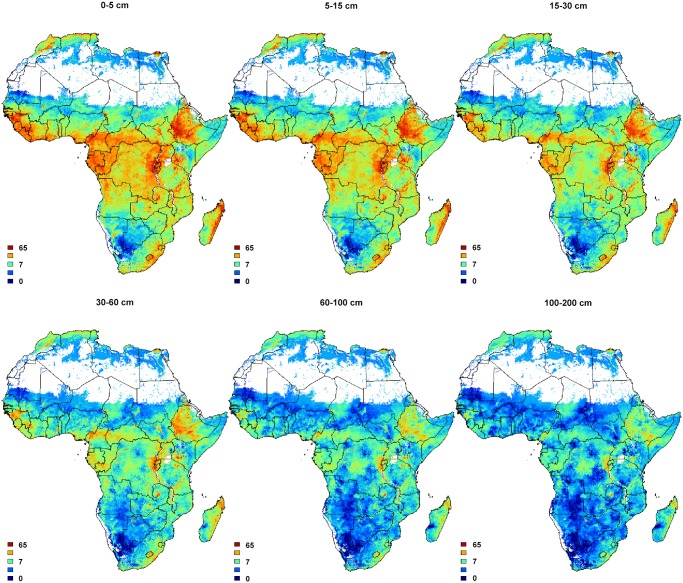
Soil organic carbon content in permilles predicted using 3D random forests RK at six standard depths. White pixels indicate excluded areas (water bodies and deserts).

**Fig 8:**
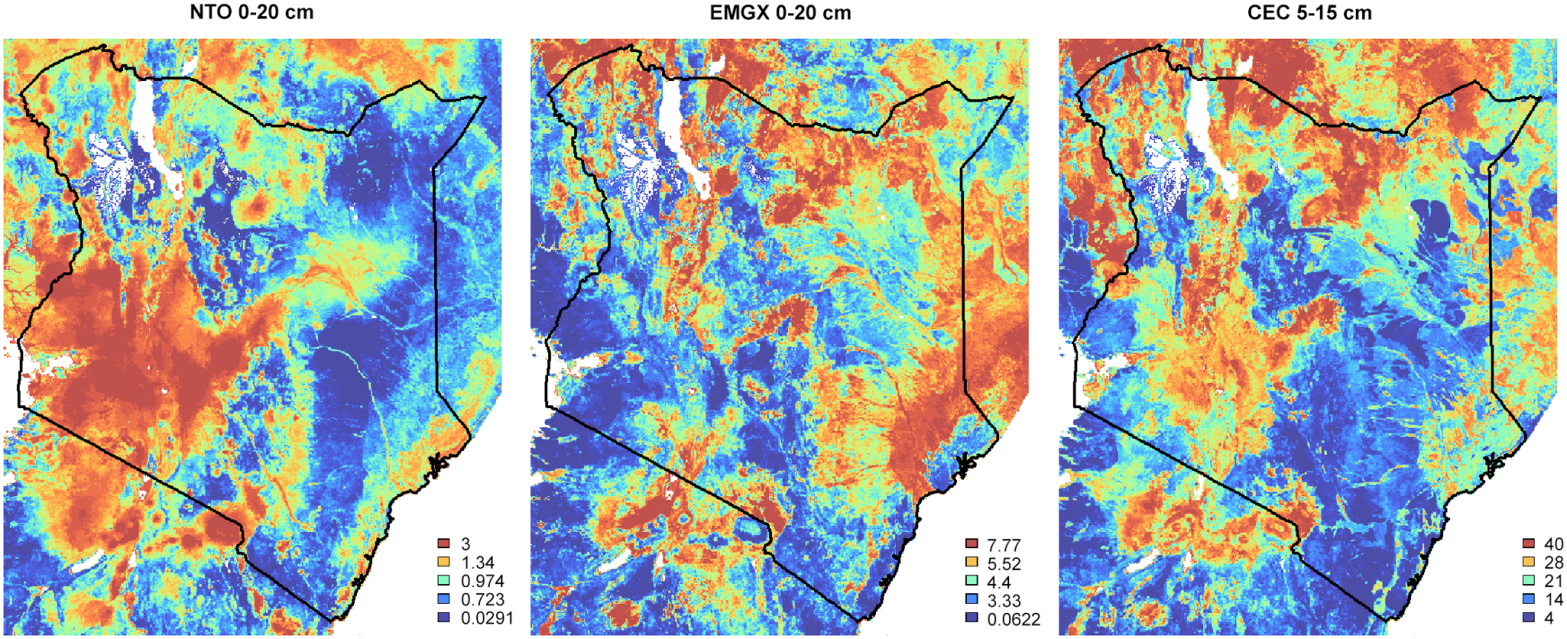
Spatial patterns of predicted total nitrogen (in permilles), exchangeable Mg (cmol+/kg) and CEC (cmol+/kg) for topsoil for Kenya. Legends were set using equally spaced quantiles. White pixels indicate excluded areas (water bodies and deserts). Each soil property is modelled independently and can thus show quite different spatial patterns.

**Fig 9:**
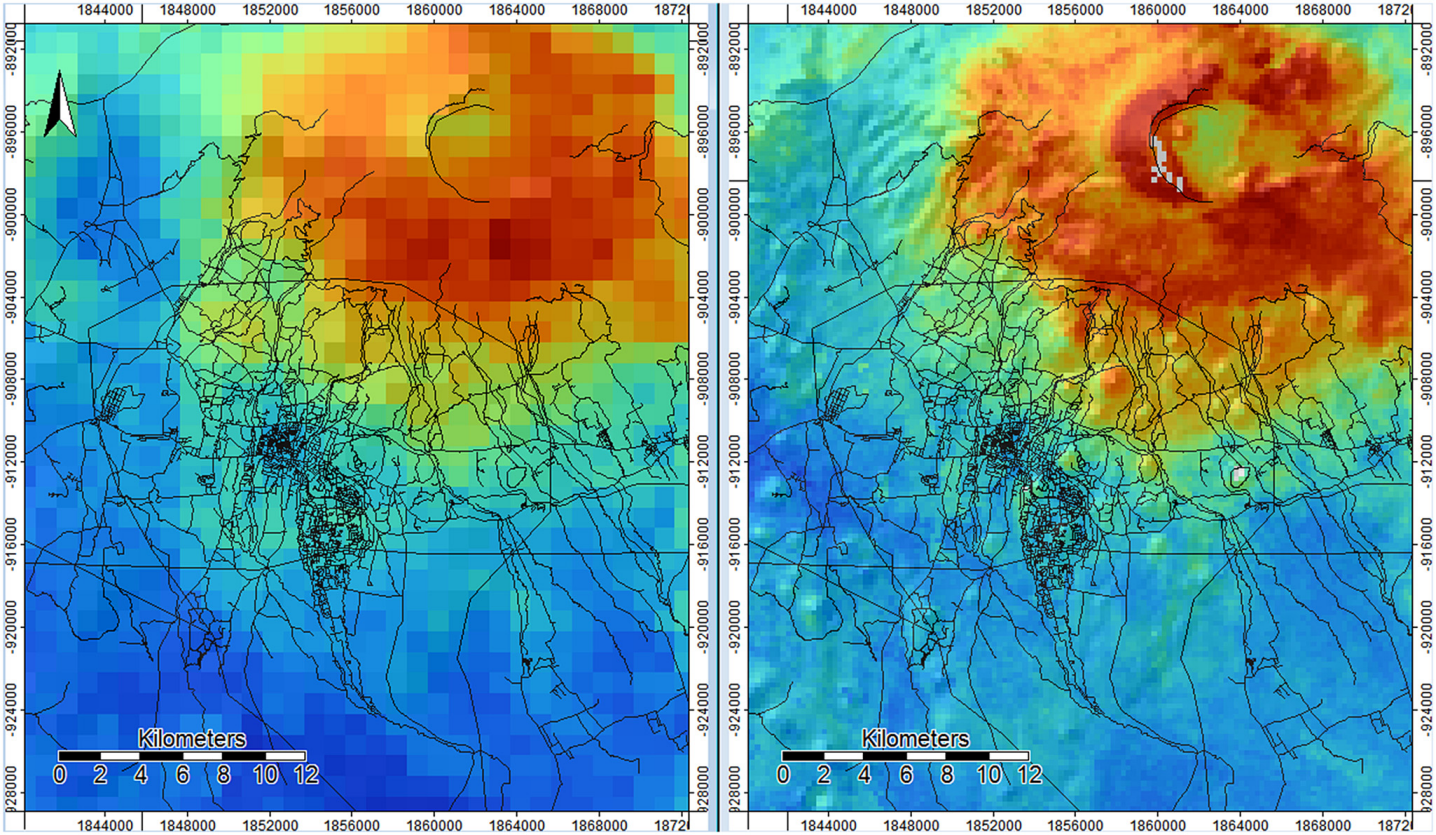
Predicted soil organic carbon content in permilles (depth: 0–5 cm) with a zoom-in on the area around the town of Arusha (Tanzania). (left) original SoilGrids1km layer at 1 km resolution vs (right) downscaled spatial predictions at 250 m resolution. Vector lines data source: OpenStreetMap.

Figs 6, 7, 8 and 9 show output predictions for soil organic carbon, total N, sum of exchangeable bases and CEC and exchangeable Mg. Fig 9 illustrates broadly consistent spatial patterns between maps at 1 km (SoilGrids1km) and 250 m (AfSoilGrids250m). The 250 m resolution predictions based on random forests models and a combination of local and global covariates exhibit, however, significantly higher mapping accuracy, i.e. an average increase of approximately 30% (see Table 2), and a 16 times greater spatial detail (from 1 km to 250 m resolution).

## Discussion

We presented initial results of mapping a selection of soil properties for the African continent by comparing and contrasting random forests regression combined with kriging to linear regression kriging. Our primary motivation for this research was to contribute to the continuous improvement of continent-wide [18, 49] and global soil mapping initiatives [21]. By using globally predicted soil properties and classes (SoilGrids1km) in combination with local covariates available for Africa and random forests regression, we tried to identify ways to improve the mapping accuracy and resolution of initial SoilGrids1km maps without requiring additional large investments. For a comparison, typical costs of soil mapping in the USA are about $1.50 USD per acre i.e. about $3 USD per ha [50], which means that, to map 21 million square-kilometers of usable land in Africa would cost approximately $6.3 billion USD following the USDA mapping standards. Estimated total costs of the USDA mapping standard include costs of field work, laboratory data analysis and generation and distribution of maps and reports. Even if we would use a more conservative estimate of the mapping costs ($8 USD per square-kilometer [51]), which better matches the resolution of 250 m, the total costs would still exceed hundreds of millions of USD. In comparison, the total budget of the AfSIS project was about 22 million USD, from which only 30–50% was used for producing soil property maps. Such a significant reduction of mapping costs per area was only possible because we focused on utilizing:

1. existing legacy soil profile data, rather than new soil surveys;
2. new inexpensive measurement technologies, such as soil spectroscopy, to reduce the costs of measuring the soil properties in laboratory;
3. publicly available remote sensing based covariate layers such as MODIS and SRTM DEM derivatives; and
4. free and open-source software for data analysis and visualization.

We evaluated the mapping performance of the two approaches using 5–fold cross-validation (see Table 2 and Fig 4). The evaluation showed an increase in mapping accuracy between 15–75% as a result of using random forests and finer-resolution covariates in addition to coarse resolution global soil maps. Thus, we believe that the additional time spent on model fitting and preparing all covariates for use in random forests modelling is worth the extra effort. This is in line with Van Ranst et al. [3], who specifically suggested that: “*The (soil survey) process should be cost-effective and economic, and the product more precise and accurate, less cumbersome for manipulations, and more amenable for use by a variety of disciplines*.”

The use of random forests improved the mapping accuracy for all soil properties when compared with linear regression, but Table 2 and Fig 4 show that there are still large prediction errors and considerable scatter around the 1:1 line. We should note here that the results of the cross-validation only give an indication of the true accuracy of the prediction maps because not all sampling locations were collected using probability sampling [52]. For this reason, we decided to focus on cross-validation of the regression part only. If we would have included kriging in the cross-validation, this would have likely resulted in more optimistic *ME* and *RMSE* simply because the points are highly spatially clustered (Fig 3).

A large portion of variation in soil properties will likely remain unpredictable. As mentioned previously, this is because the legacy soil profile data are a compilation of various data sets with significant measurement and positional errors. Legacy soil profile data are also largely outdated (Fig 1). On the other hand, response to nutrient applications is largely driven by soil water availability, which is largely dependent on properties that are stable in time (soil texture and coarse fragments), making legacy soil data still cost-effective. The majority of sentinel site soil property data were derived from soil spectroscopy data and these also introduce uncertainties. It is also clear from Fig 4 that, especially in the case of organic carbon and clay content, high values are underpredicted while low values are overpredicted. This is due to the well-known smoothing effect of regression and kriging.

Although the random forests algorithm turns out to be a superior spatial prediction framework, a drawback of using random forests is that these can take considerably more time to fit and make predictions than linear regression. In our case it took on average 30–60 min to fit a random forests model per property (see Table 1) and about 10 days of continuous computing to produce all prediction maps (on a 12–core HP Z420 workstation with 64 GiB RAM running on a Windows 7 64-bit system). Just for illustration, one image of Africa at this resolution is 29,501 columns by 31,505 rows or about 300–600 MiB after (gzip) compression (the soil mask map of Africa contains a total of 331 million pixels / prediction locations). To produce all predictions of all properties at six standard depths requires almost 1 TiB of storage space. Hence further improvements will need to focus on optimizing the computing (e.g. moving to cloud computing) and speeding up reading/writing of the soil images (e.g. reading and writing from the R environment to final output GIS formats).

**Fig 10:**
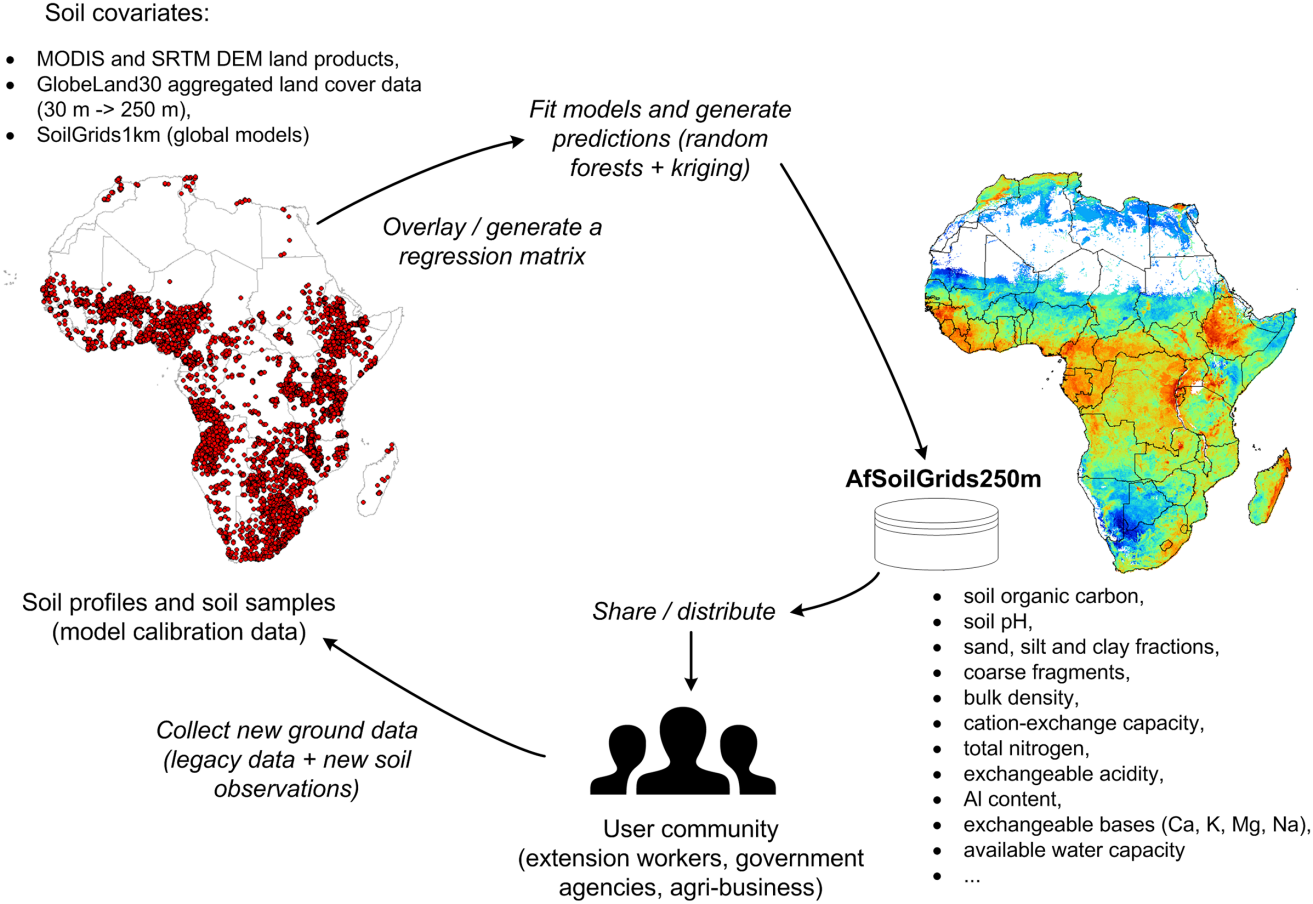
Spatial prediction scheme used to produce AfSoilGrids250m data. Spatial predictions in the case of an automated soil mapping system can be continuously updated by adding new soil field observations and new covariates.

Validating spatial predictions for the entire continent of Africa is certainly not trivial and probably requires a collaborative effort of all countries involved. It is clear from Fig 2 that many countries, especially in the northern part of Africa, but also large land areas such as the Democratic Republic of Congo, Namibia, Chad and South Sudan, are under-represented with only few profiles available for vast areas (see also Fig 3). In many countries in Africa there are, unfortunately, security issues that restricted our ability to collect both new Sentinel Site data and existing legacy data. In that sense, these initial spatial predictions should be considered only a beginning of an ongoing process of automated soil mapping of the African continent (Fig 10).

The *F*-test results in Table 2 showed that the accuracy improvements were highly statistically significant for all properties. This is partially because with a very large number of soil samples (see Table 1) even a small difference in variance (and *RMSE*) between two methods can be statistically significant. In that context, Δ*RMSE*_%_, i.e. the relative improvement in the *RMSE*, is probably more informative for judging the added value of non-linear regression and local covariates, while *F*- and *t*-tests are only useful to rule out that prediction improvements are due to chance effects. This study showed that use of random forests for modelling all soil properties, but especially for coarse fragments and nitrogen content, is simply a better choice than linear regression.

Because exchangeable Mg, Ca, Na, K and total bases are highly correlated, their prediction maps and importance plots are also similar. Aluminium, on the other hand, is slightly negatively correlated with the cations, implying that the map of Aluminium concentration has a reversed spatial pattern (Fig 6). This is expected as strongly weathered soils tend to have higher Aluminium and lower base status. However, many areas with high concentration of exchangeable bases in Africa are deficient in nitrogen and available phosphorus and possibly have acidity or toxicity problems. This implies that delineating the most fertile soils in Africa also requires information derived from additional soil property maps e.g. organic carbon and nitrogen content maps. Furthermore, we should emphasize that maps based on soil legacy data (Fig 2) may not accurately depict the current status of soil properties, which may have undergone large changes over time due to changes in land use and management. While our maps provide a useful overview of soil fertility status in Africa, only recently (and properly) collected, new soil samples are suitable for establishing relevant baselines for soil health surveillance [53].

**Fig 11:**
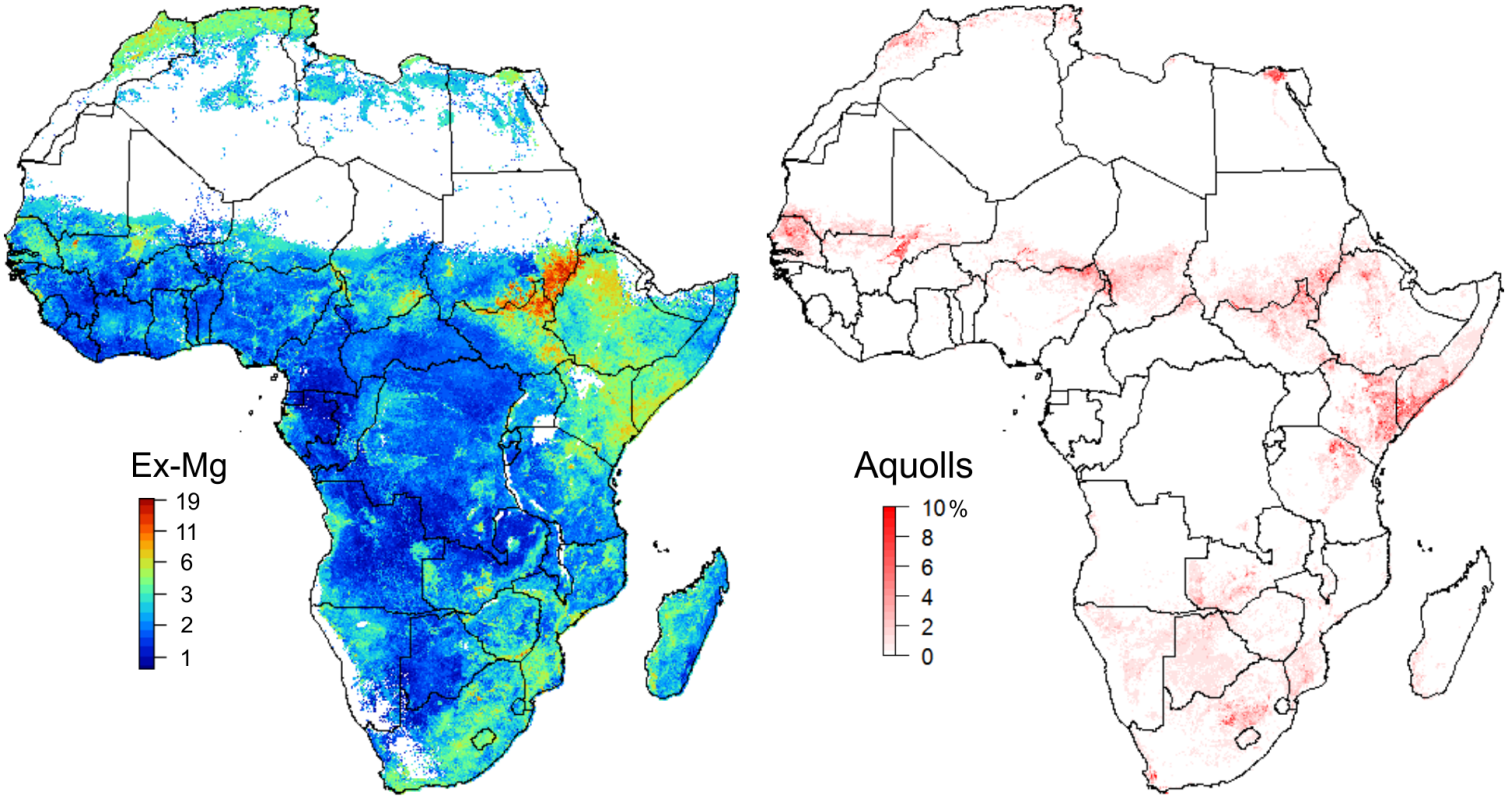
High values of exchangeable bases in Africa coincide with the predicted distribution of Alfisols and Mollisols. distribution of Aquolls from SoilGrids1km [21] (right) and the predicted exchangeable Mg (left). See also Fig 5.

**Fig 12:**
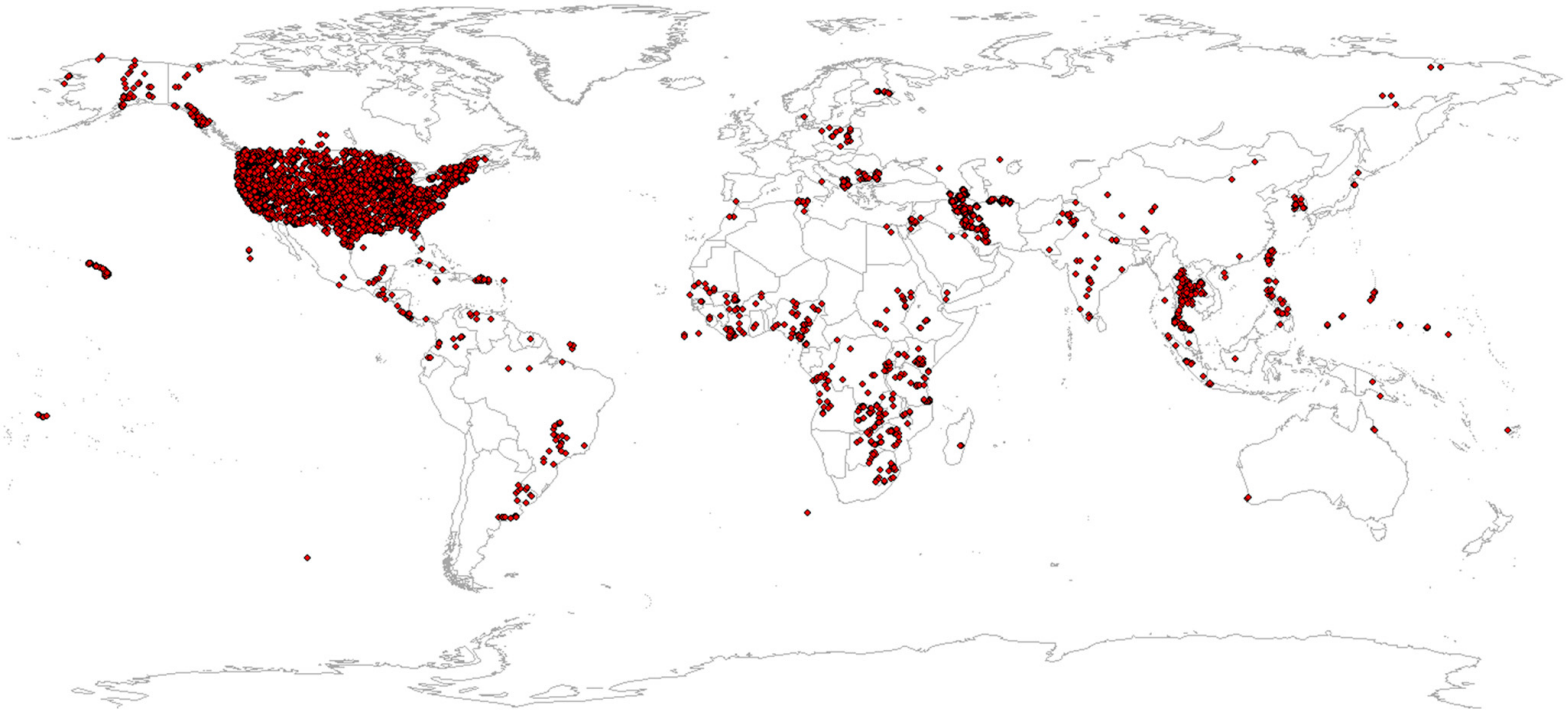
Global distribution of profiles with observed USDA Soil Taxonomy class. observations used as calibration data for producing SoilGrids1km predictions [21]. The majority of observations (> 80%) come from the USA National Cooperative Soil Survey Soil Characterization database.

An interesting discovery of our investigation is that globally predicted Soil Taxonomy suborders significantly help improve predictions of exchangeable elements (Figs 5 and 11). Knowing that there are only about 2500 soil profiles that provide information on Soil Taxonomy classes in Africa (Fig 12) it appears that there is indeed an added value in using soil classes as covariates. Note that calibration of the regression models used to derive the global SoilGrids1km soil map was able to make use of a much larger global soil data set (ca. 28,000 profiles with USDA Soil Taxonomy classification, with > 80% of these from the North American continent; Fig 12). These findings draw attention to the potential of using calibration data and soils knowledge obtained from densely sampled countries to improve mapping of soil in countries with limited soil data. Our results also suggest that soil mapping projects can benefit substantially from including a relatively large number (e.g. 10–100) of inexpensive soil class observations for every soil profile that is analysed in the laboratory.

Although the global soil covariates (SoilGrids1km maps) proved very useful, it should be mentioned that these were derived from numerous global covariate layers that could equally have been included in mapping directly. Theoretically, the prediction accuracy can be improved using the original global covariates, because it is a more flexible approach that does not require that all covariate information must be passed through a SoilGrids1km map. However, from a practical point of view there is much value in using an existing global soil map directly as a covariate in regional-scale mapping. It simplifies the model building process while still capturing the most important information. The pros and cons of various approaches should be tested in future research, also for mapping soil properties at the national scale.

We need to emphasize that these are preliminary results and output maps will be re-computed and gradually improved until stable results are produced, as was the case with the SoilGrids1km maps [21]. We have approached a level of spatial resolution that could prove useful for regional to sub-regional planning, but is probably not yet sufficiently detailed for site level planning or operational management. Nevertheless, the output raster maps exhibit clearly improved spatial detail and are more accurate than the previous 1 km resolution maps (see e.g. Fig 9). Future applications will need to evaluate the actual benefit / gain that is realized by using this soil information for agricultural planning and decision making. Another future research direction would be to develop methodologies to allow for multi-scale merging of spatial predictions of soil properties from national or local initiatives.

There are also other limitations to automated soil mapping, i.e. machine learning algorithms for producing soil information, that need to be mentioned here. Firstly, the number of accurately georeferenced locations of reliable soil observations (particularly with analytical data) is often not sufficient to completely capture and describe all patterns of soil variation in an area. There may be too few sampled points and the exact location of point data may not be correctly recorded. In short, data-driven soil mapping is field-data demanding and collecting field data requires significant efforts. Secondly, there is no guarantee that the available soil point data, apart from the AfSIS Sentinel Site data, are truly representative of the dominant patterns and soil forming conditions in Africa. Many traditional soil survey points are selected and sampled purposely to locate soil mapping unit boundaries and hence transition areas are overrepresented [54]. Soil surveyors often also sample with an obvious bias towards potentially productive areas. In the case of the AfSP and AfSS datasets, it seems that especially tropical soils and desert soils have been heavily under-represented. We encourage national and regional agencies to take a critical look at the prediction maps and help us improve these by providing local data, local validation reports or quality checks.

In summary from all analyses shown, two main findings can be emphasized:

1. Random forests proved to be a more successful prediction method than multiple linear regression with an average improvement in mapping accuracy (Δ*RMSE*_%_) of about 20%. Fitting and running random forests models takes an order of magnitude more time and the modelling success is sensitive to artifacts in the input data, but as long as quality-controlled point data are provided, an increase in soil mapping accuracy can be expected.
2. SoilGrids1km global predictions can help produce more accurate predictions of soil properties in Africa when these are refined with finer-resolution information derived from a DEM or satellite imagery. Global and local covariates can be elegantly combined to produce globally consistent and complete soil maps.

## Supporting Information

S1 Regression-kriging in R using the Meuse data set Regression-kriging and comparison of spatial prediction efficiency explained using using the Meuse data set [30]. For more R code examples please also refer to the GSIF tutorial at http://gsif.isric.org.
(PDF)
