# Supplementary material for: Mapping Soil Properties of Africa at 250 m Resolution: Random Forests Significantly Improve Current Predictions
Source: PLoS One. 2015 Jun 25;10(6):e0125814. doi: 10.1371/journal.pone.0125814 (PMC4482144; doi:10.1371/journal.pone.0125814)
Supplement: S1 Regression-kriging in R using the Meuse data set — For more R code examples please also refer to the GSIF tutorial at http://gsif.isric.org. (PDF) [file pone.0125814.s001.pdf]

Mapping soil properties of Africa at 250 m resolution: random forests significantly improve current predictions

Tomislav Hengl[1,*], Gerard B.M. Heuvelink[1], Bas Kempen[1], Johan G.B. Leenaars[1], Markus G. Walsh[2], Keith Shepherd[3], Andrew Sila[3], Robert A. MacMillan[4], Jorge Mendes de Jesus[1], Lulseged Tamene[5], Jérôme E. Tondoh[3]

1 ISRIC — World Soil Information, Wageningen, the Netherlands

2 The Earth Institute, Columbia University, USA / Selian Agricultural Research Inst., Arusha, Tanzania

3 World Agroforestry Centre, Nairobi, Kenya

4 LandMapper Environmental Solutions Inc., Edmonton, Canada

5 International Center for Tropical Agriculture, Lilongwe, Malawi

∗ **E-mail: tom.hengl@wur.nl**

Supporting Information

S1 Regression-kriging in R using the Meuse data set

Consider the Meuse data set from the sp package in the R environment for statistical computing, which is commonly used to illustrate various geostatistical mapping steps [1]. By using the generic `fit.gstatModel` function from the GSIF package (Global Soil Information Facilities) we can predict soil organic matter using linear regression-kriging with:

```
R> library(sp)
R> library(gstat)
R> library(GSIF)
R> set.seed(2419)
R> demo(meuse, echo=FALSE)
R> omm1 <- fit.gstatModel(meuse, om~dist+soil, meuse.grid, family = gaussian(log))
```

```
Fitting a linear model...
Fitting a 2D variogram...
Saving an object of class 'gstatModel'...
```

where `fit.gstatModel` wraps up regression modelling and fitting of the residual variogram, and creates an object of class `gstatModel` that consists of the: (1) regression model [2], (2) residual variogram model, and (3) spatial locations of observations used to fit the model. Note

that above it is assumed that soil organic matter is lognormally distributed, and that it is exponentially related to distance to river (*dist*) and soil type (*soil*). Running GSIF function `predict` on this object will produce predictions using the regression-kriging framework, and will run a 5–fold cross-validation:

```
R> rk1 <- predict(omm1, meuse.grid)
```

Within the GSIF package, the switch to random forests RK is achieved by changing the `method` argument:

```
R> omm2 <- fit.gstatModel(meuse, om~dist+soil, meuse.grid, method = "randomForest")
```

which fits a random forests model, as implemented in the randomForest package [3].

We can now determine if there is a significant improvement in the mapping accuracy, by computing the Mean Error (*ME*), which is a measure for prediction bias, and the Root Mean Squared Error (*RMSE*), which is a measure for the random prediction error. These measures are computed from the predicted and observed values at cross-validation points [4]:

```
R> rk2 <- predict(omm2, meuse.grid)
R> mean(rk1@validation$observed - rk1@validation$var1.pred)

[1] 0.01460599

R> summary(rk1)$RMSE

[1] 2.472

R> mean(rk2@validation$observed - rk2@validation$var1.pred)

[1] -0.002261916

R> summary(rk2)$RMSE

[1] 2.077
```

Note that validation is repeated here 5 times (5–fold cross-validation) to account for chance effects — the best algorithm can change by even removing an outlier or choosing different validation data even within the same data set. In the case above, results show that both methods produce a *ME* close to zero, indicating that both methods are unbiased. In addition, the random forests RK model has an $RMSE = 2.077$ (61 % of total variance in organic matter explained by the model) compared to $RMSE = 2.472$ (47 % of total variance explained) obtained with the linear RK model, which is an improvement of approximately 15 %.

Assuming that the prediction locations are a random sample of the study area (which, in fact, we know they are not), we can also test if the random forests predictions are significantly more accurate than the linear RK predictions, using standard t- and F-tests [5]:

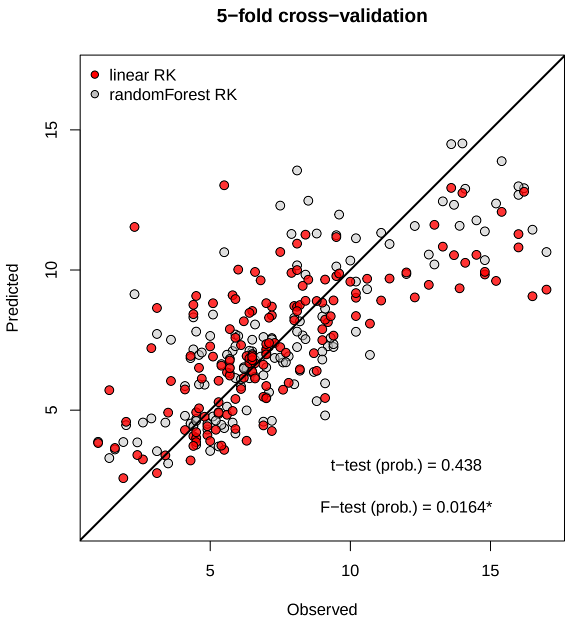

Figure 1. Comparison of prediction performance for predicting soil organic matter (Meuse data set) assessed using 5–fold cross-validation: comparison of performance for linear RK (red) vs random forests RK (grey). P-value of F-test indicates that random forests RK cross-validation errors have a significantly smaller variance than linear RK cross-validation errors. See text for more explanation.

```
R> res1 <- rk1@validation$residual
R> res2 <- rk2@validation$residual
R> t_test <- t.test(res1, res2, alternative = "greater", paired = TRUE)
R> v_test <- var.test(res1, res2, alternative = "greater")

R> t_test$p.value

[1] 0.437643

R> v_test$p.value

[1] 0.01640839
```

The t-test (`t.test`) evaluates whether the two methods have the same mean error (*ME*), while the F-test (`var.test`) evaluates whether the methods have the same variance, i.e. the same *RMSE*, assuming that the *ME*'s are the same. Note that here we used one-sided tests in order to examine whether or not random forest performs more accurately than linear regression.

The test results show that the variance differences are significant at the 5 % level (i.e. the residual variance of the linear RK model is significantly larger than that of the random forests

RK model), while the differences between the *ME*'s are not statistically significant ($p > 0.05$, hence the null hypotheses of equal means cannot be rejected). Hence we conclude that the extension from linear regression to machine learning leads to a statistically significant improvement of mapping accuracy at the 5 % level (see also Figure 1). The t-test shows that both methods perform equally well in terms of the *ME*. Both turn out to be not-significantly different from zero, which indicates that both prediction methods provide unbiased predictions of the soil property.

References

1. Bivand R, Pebesma E, Rubio V. Applied Spatial Data Analysis with R. 2nd ed. Use R Series. Heidelberg: Springer; 2013.
2. Hastie TJ. Generalized additive models. In: Chambers JM, Hastie TJ, editors. Statistical Models in S. Wadsworth & Brooks/Cole; 1992. p. 249–307.
3. Liaw A, Wiener M. Classification and Regression by randomForest. R News. 2002;2(3):18–22. Available from: http://CRAN.R-project.org/doc/Rnews/.
4. Hengl T, Nikolić M, MacMillan RA. Mapping efficiency and information content. International Journal of Applied Earth Observation and Geoinformation. 2013;22:127–138.
5. Snedecor GW, Cochran WG. Statistical Methods. 8th ed. Iwoa State University Press; 1989.
